# Dynamics of Matricellular Protein Levels in Blood Predict Recovery in Patients with Human Immunodeficiency Virus-Tuberculosis Coinfection

**DOI:** 10.3390/v16050664

**Published:** 2024-04-24

**Authors:** Ashwini Shete, Manisha Ghate, Hiroko Iwasaki-Hozumi, Sandip Patil, Pallavi Shidhaye, Gaowa Bai, Takashi Matsuba, Pratiksha Pharande, Bharati Mahajan, Aarti Randive, Anupam Mukherjee, Toshio Hattori

**Affiliations:** 1 Indian Council of Medical Research—National Institute of Translational Virology and AIDS Research (ICMR-NITVAR, Formerly National AIDS Research Institute), Pune 411026, India; ashete@nariindia.org (A.S.); mghate@nariindia.org (M.G.); spatil@nariindia.org (S.P.); pshidhaye@nariindia.org (P.S.); pratiksha.pharande1901@gmail.com (P.P.); bmahajan.nari@gov.in (B.M.); randiveaarti83@gmail.com (A.R.); amukherjee@nariindia.org (A.M.); 2 Research Institute of Health and Welfare, Kibi International University, Takahashi 716-0018, Japan; hiro_ihz@kiui.ac.jp; 3 College of Food Science and Engineering, Inner Mongolia Agricultural University, Hohhot 010018, China; bgw2291@imau.edu.cn; 4 School of Pharmaceutical Science, Kyushu University of Medical Sciences, Nobeoka 882-8508, Japan; matsubat@phoenix.ac.jp; 5 Shizuoka Graduate University of Public Health, Shizuoka City 420-0881, Japan

**Keywords:** pulmonary tuberculosis, extrapulmonary tuberculosis, HIV, osteopontin, galectin-9

## Abstract

Chronic immune activation in tuberculosis (TB) associated with human immunodeficiency virus (HIV) infection (HIV/TB) modifies their clinical course. We prospectively measured osteopontin (OPN), full-length galectin-9 (FL-Gal9), and total-Gal9 (T-Gal9) levels in 32 patients with HIV/TB coinfection treated with anti-tuberculosis and antiretroviral therapies over 6–18 months to determine the amelioration of inflammatory conditions in response to the therapies. We observed a significant time-dependent decrease in FL-Gal9 in both pulmonary TB (PTB, n = 20) and extrapulmonary TB (EPTB, n = 12) patients. The levels of T-Gal9, OPN, and CRP decreased significantly after treatment in only PTB patients. We calculated the inflammatory score (INS) indicating immunologic recovery based on the decline in OPN, FL-Gal9, T-Gal9, and CRP levels. Baseline levels of T-Gal9 and OPN positively correlated with INS in all TB and only PTB patients, respectively, indicating that their levels predict better recovery. In contrast, FL-Gal9 levels at the second visit negatively correlated with INS in EPTB patients. The decrease rate in OPN levels at the second visit also correlated positively with INS in PTB patients. Women showed a higher INS and lower levels of FL-Gal9 than men. The patients with moderate grade severity on chest X-ray had higher CD4 cell numbers than those with limited grade severity. Monitoring these markers will help to predict and assess the response to therapy as well as to devise strategies to reduce the complications caused by chronic immune activation in patients with HIV/TB coinfection.

## 1. Introduction

Human immunodeficiency virus (HIV) infection is the most potent risk factor for developing tuberculosis (TB) by weakening immune responses against Mycobacterium tuberculosis (MTB) [1,2]. Notably, compared to the general population, the risk of active TB is 19% higher in people living with HIV (PLHIV) and remains higher despite their being prescribed effective ART [3], although the incidence reduces considerably with the duration of ART [4]. HIV-induced immunosuppression modifies the clinical presentation of HIV/TB coinfection [5]. PLHIV are less likely to present with pulmonary TB even if they have CD4+ T cell counts in a normal range. Contrarily, they commonly present with extrapulmonary manifestations such as pleural effusion or lymphadenopathy. PLHIV with advanced disease show atypical signs and symptoms and more frequent extrapulmonary dissemination [6]. They can present with all varieties of extrapulmonary TB (EPTB) involving various organs like the bone, liver, spleen, vertebrae, meninges, spine, and kidney. More than 50% of patients with HIV/TB coinfection are reported to present with extrapulmonary manifestations [7]. The treatment for HIV/TB coinfection is also challenging [8]. The standard 6-month regimen results in the prompt sterilization of sputum and low treatment failure rates, similar to those observed in HIV-negative persons [9]. However, studies have documented higher rates of relapse in patients with HIV/TB coinfection who received anti-TB therapy (ATT) for 6 months, as compared with 9–12 months [10]. Scores based on the expression of interferon-stimulated genes (ISG) at baseline and post-treatment showed a modest prognostic performance for recurrent TB, indicating the role of inflammatory markers and immunological recovery in predicting TB recurrence [11].

HIV-1 infection causes a chronic inflammatory condition leading to immunodeficiency [12]. Galectin-9 (Gal-9) and Osteopontin (OPN) are matricellular proteins (MCPs) involved in inflammation and extracellular remodeling by acting as connective tissue growth factors. The plasma levels of both Gal-9 and OPN are elevated in various infectious diseases [13,14]. We reported that plasma Gal-9 levels were shown to have the potential to serve as a cheaper surrogate marker of viremia in HIV-positive patients on ART with a very high sensitivity and specificity [15]. Another group suggested that elevated Gal-9 levels are also predictive of deleterious non-acquired-immunodeficiency-syndrome (non-AIDS) events, particularly cardiovascular complications, in people with HIV initiating ART [16]. On the other hand, OPN levels reflect the acute response and severity of MTB infection [17,18]. Interestingly, the levels of OPN protein expression were inversely correlated with disseminated infection and the patient’s death. Therefore, it was proposed that OPN contributes to human resistance against mycobacteria [19]. Recently, gene therapy using an adenovirus vector encoding the OPN gene led to high immune responses against TB infection in mice [20]. 

When evaluating Gal-9 and OPN functions, we need to remember that Gal-9 and OPN are highly susceptible to proteolysis via various biological reactions, including inflammation and resistance against infections. The proteolysis is supposed to erase or change the bioactivities of Gal-9 and OPN [13,14]. Therefore, we need to clarify whether (1) full-length (FL), (2) cleaved, or (3) both FL and cleaved forms of both Gal-9 and OPN were examined when measuring their circulating levels. Gal-9 is separated into N- and C-terminal carbohydrate-recognition domains (NCRD and CCRD, respectively) by proteolysis in the linker peptide of the molecule [21]. We reported that the plasma levels of Tr-Gal9 measured by Galpharmer Elisa and T-Gal9 measured by R&D Elisa are the most sensitive biomarker of inflammation and severity in AIDS and HIV/TB coinfection [22,23]. Similarly, we measured the total plasma concentration of FL and the cleaved form of OPN by R&D Elisa [14,24]. Their levels were reported to help in monitoring the severity of HIV/TB coinfection in a cross-sectional analytical study [21,22,23]. 

For the clinical treatment of HIV/TB coinfection, it is important to manage the prognosis of the patients. In this study, we monitored the time course of the plasma levels of T-Gal9, FL-Gal9, and OPN in AIDS patients with pulmonary TB (PTB) and EPTB. Moreover, we assessed the degree of inflammation at the last hospital visit in each of those patients as a single numerical value that was expressed as inflammatory score (INS). Through these analyses, we clarified that measuring plasma FL-Gal9, T-Gal9, and OPN levels at the initial stage of treatment can predict the recovery of inflammation at the end of therapy. We aimed to clarify that the monitoring of plasma Gal-9 and OPN could help the management of patients with HIV/TB coinfection in the prospective study.

## 2. Materials and Methods

### 2.1. Study Settings and Participants

**Figure 1:**
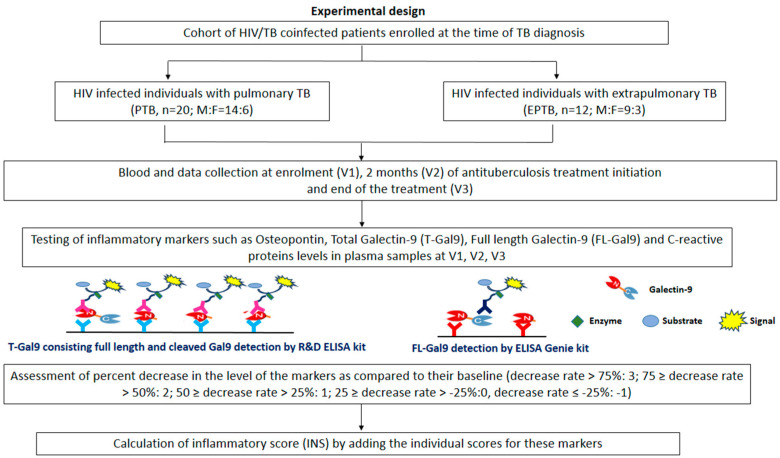
Flow chart of the experimental design: The figure explains the flow of events in terms of the number of enrolled patients, longitudinal follow-up, and the calculation of inflammatory score. It also explains the methodology used for detecting total Galectin-9 and full-length Galectin-9 in plasma samples by different ELISA kits.

This prospective study was conducted in four ART centers in Pune, India. Adult HIV-positive individuals, co-infected with TB and either to be initiated or within 14 days of anti-tubercular treatment initiation, were enrolled. Individuals with PTB were microbiologically confirmed as having tested positive by sputum acid-fast bacillus test (AFB) and/or Xpert MTB/RIF assay. The diagnosis of EPTB was primarily presumptive and was based on clinical signs and symptoms, as well as radiological investigations. The spectrum of EPTB patients comprised lymphadenopathy (6), abdominal tuberculosis (5), and meningitis (1). The experimental design is explained in Figure 1.

### 2.2. Sample Collection and Data Collection

Blood samples were collected at baseline (V1), at the second month (V2), and at the end of treatment (V3). Plasma samples were separated and stored at −80 °C until testing. Sociodemographic and clinical data were collected using a structured questionnaire and included clinical, radiological, and laboratory findings. 

### 2.3. Estimation of Matricellular Proteins and Other Inflammatory Markers

Inflammatory conditions of the patients were monitored by measuring the plasma concentration of OPN, T-Gal9, FL-Gal9, and CRP. The values were determined by commercially available ELISA or Luminex assay at the three aforementioned time points as described previously [23].

#### 2.3.1. OPN ELISA Assay

Human Osteopontin DuoSet ELISA Kits (R&D Systems, Minneapolis, MN, USA) were used to measure plasma concentrations of OPN. The manufacturer’s instructions were followed to process the plasma samples (dilution: 1:100) for the detection of OPN. The limit of detection (LOD) for the OPN ELISA was 62.5 pg/mL. The kits can detect both FL and cleaved forms of OPN [24].

#### 2.3.2. T-Gal9 ELISA Assay

Human Galectin-9 DuoSet ELISA Kits (R&D Systems, Minneapolis, MN, USA) were used to measure plasma concentrations of T-Gal9. The manufacturer’s instructions were followed to perform the ELISA after diluting plasma samples to 1:10 through a reagent diluent supplied with the kit. The limit of detection (LOD) for the T-Gal9 ELISA was 93.8 pg/mL. The kits can detect both FL and cleaved forms of Gal9 [21].

#### 2.3.3. Full-Length Galectin-9 ELISA Assay

Human GAL9 ELISA Kit (ELISA Genie, Dublin, Ireland) was used to measure the FL-Gal9 concentrations in the plasma samples of the study participants. The kit uses a capture antibody against N-CRD and a detection antibody against C-CRD of Galectin-9, and hence specifically detects FL-Gal9. The LLOD for detecting the FL- Gal9 by the kit was 7.8 pg/mL.

#### 2.3.4. CRP ELISA Assay

High-Sensitivity C-Reactive Protein Enzyme Immunoassay Test Kit (Bio Check, San Francisco, CA, USA) was used to measure the concentrations of CRP in plasma samples as per the manufacturer’s instructions. The analytical range of the ELISA was 0.1–10 mg/L. Samples with values above 10 mg/L were retested after further diluting them 10 times with the sample diluent.

### 2.4. Classification of the Severity of the Chest X-ray Findings

Limited involvement is defined as the presence of lesions with slight to moderate density, but no cavitations. Lesions may be present in a small portion of one or both lungs, but the total extent of the lesions should not exceed the size of the apex of the lung.

Moderate involvement is defined as lesions present in one or both lungs, with a total extent which does not exceed the following:
Scattered lesions of slight to moderate density that may extend throughout the total volume of one lung or may partially involve both lungs.
Dense, confluent lesions that extend to up to 1/3 of the volume of one lung. Cavitation with a diameter of <4 cm.

### 2.5. Scoring for Inflammatory Markers

INS in each patient was also calculated as follows:

For OPN, T-Gal9, FL-Gal9, and CRP levels, each decrease rate was calculated as the percentage of concentration at the first visit minus concentration at the third visit. Based on each decrease rate, the score for each marker was defined as follows: decrease rate > 75%: 3; 75 ≥ decrease rate > 50%: 2; 50 ≥ decrease rate > 25%: 1; 25 ≥ decrease rate > −25%:0; decrease rate ≤ −25%: −1. Finally, the total score, calculated by adding the individual scores for these markers, was designated as INS.
INS classified each patient into immunologic successor (above 6 for all TB patients and 8 for only PTB) and failure (below 5 for all TB patients and 7 for only PTB). The median of the total score was used to classify each patient as a successor or failure and obtain the cut-off value of each marker to discriminate successors from failures using ROC curve analysis.

### 2.6. Statistical Analysis

A statistical analysis was performed using GraphPad Prism 8 (San Diego, CA, USA). Differences between the two groups and among multiple groups were assessed by Mann–Whitney U and Kruskal–Wallis tests, respectively. Correlations were assessed by Spearman’s rank correlation coefficient. An ROC analysis was conducted to analyze the ability of biomarkers to recognize PTB and EPTB groups showing immunological recovery.

## 3. Results

### 3.1. Characterization of the Patients

We enrolled 32 participants with HIV who were diagnosed with TB at ART centers in Pune, India. Of the 32 individuals with HIV/TB coinfection, 20 and 12 were diagnosed with PTB and EPTB, respectively. PTB patients were microbiologically confirmed as having tested positive by Sputum acid-fast bacillus test (AFB) and/or Xpert MTB/RIF assay. The rifampicin-resistance type was detected in one of the PTB patients. The spectrum of EPTB patients comprised lymphadenopathy (6), abdominal kochs (5), and meningitis (1). The individuals were followed three times during their treatment period: at enrollment (V1), after two months (V2), and at the end of therapy (V3). The samples could not be collected for three individuals at the end of therapy. Of the total number of individuals with HIV-TB coinfection, 9 of 12 individuals with EPTB (75%) and 11 of 20 individuals with PTB (55%) were on antiretroviral therapy.

### 3.2. Analysis of Markers of the Patients

The baseline data of the enrolled participants are listed in Table 1. We found no significant differences in age, body weight, the duration of therapy, gender, and viral load between individuals with PTB and EPTB patients. CD4 counts were significantly higher in EPTB patients than those with PTB at the enrolment visit as a higher proportion of EPTB patients were on ART than the PTB patients. The levels of FL-Gal9 (one tailed *p* value = 0.0305) and CRP (*p* = 0.0081) were found to be significantly lower in individuals with EPTB than those with PTB at baseline. OPN and T-Gal9 levels did not differ significantly at baseline in individuals with PTB and EPTB. An inflammatory score (INS) was devised to express the decline in these markers in comparison to their baseline levels. A higher score indicated a greater degree of recovery from inflammation as the decline in the levels was greater. The INS did not differ significantly in individuals with PTB and EPTB, although the scores tended to be higher in individuals with PTB.

**Figure 2:**
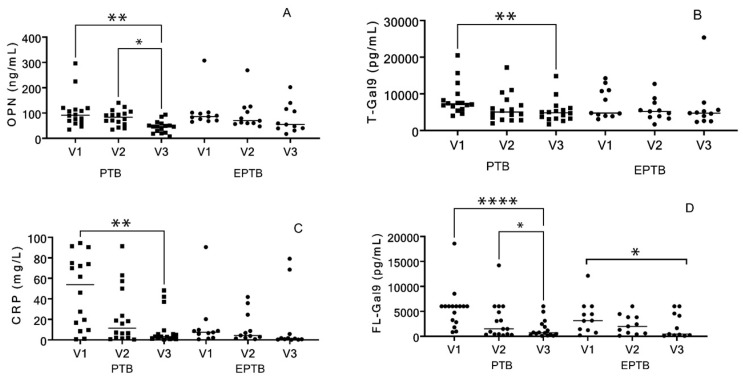
Changes in four markers during three visits. V1, V2, and V3 are the 1st, 2nd, and 3rd visits. Dot plots showing changes in (**A**) plasma OPN levels, (**B**) plasma T-Gal9 levels, (**C**) plasma CRP levels, and (**D**) plasma FL-Gal9 levels in PTB and EPTB patients at V1 (baseline), V2 (month 2), and V3 (end of treatment). *p* values showing significant changes in the levels are indicated as * (*p* < 0.05), ** (*p* < 0.01), **** (*p* < 0.0001).

All four markers (OPN, T-Gal9, FL-Gal9, and CRP) significantly decreased in PTB patients (*p* = 0.0023, 0.0023, <0.0001, and 0.0023, respectively) at the end of the treatment as compared to their baseline levels and the decrease was most remarkable for FL-Gal9. Among individuals with EPTB, the decrease was significant in FL-Gal9 only (*p* = 0.0042) (Figure 2). 

### 3.3. Correlation of Baseline Levels of Inflammatory Markers with Each Other and with INS

**Figure 3:**
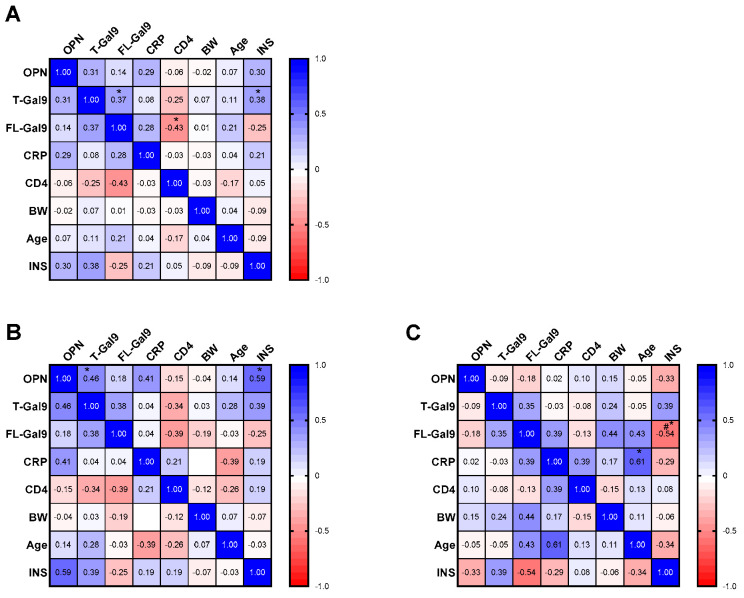
Correlations between each parameter at first visit and INS in (**A**) all AIDS/TB, (**B**) AIDS/PTB, and (**C**) AIDS/EPTB patients. The correlation coefficient is shown in each cell and as colors ranging from blue to red. OPN: osteopontin; T-Gal9: total galectin-9; FL-Gal9: full-length galectin-9; CRP: C-reactive protein; BW: body weight; INS: inflammatory score, * *p* < 0.05; #* *p* < 0.05 (one-tailed).

**Figure 4:**
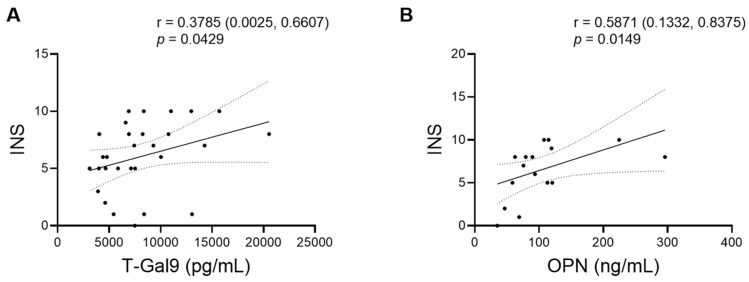
Correlations of INS with markers at the first visit. Scatter plots for positive correlations of INS with (**A**) plasma T-Gal9 levels in all TB patients and with (**B**) plasma OPN levels in PTB patients are shown accompanied by correlation coefficients (*r*) with 95% confidence interval (CI) and *p*-value. 95% CI is also shown by dotted ranges.

We investigated whether the levels of the parameters at the first visit were related to each other and INS. Notably, INS correlated differentially with different markers. We found a positive correlation of T-Gal9 levels with INS in all TB patients, irrespective of the site involved (Figure 3A and Figure 4A). Conversely, a negative correlation was observed between FL-Gal9 levels and INS in individuals with EPTB (*p* = 0.0371, one-tailed; Figure 3A), while INS was found to correlate positively with OPN in individuals with PTB (Figure 3B and Figure 4B). We also found a positive correlation of T-Gal9 and FL-Gal-9 levels in individuals with TB, irrespective of the site of infection, while individuals with PTB showed a positive correlation between T-Gal-9 and OPN (Figure 3B). Additionally, we found a negative correlation of FL-Gal9 levels with CD4 counts in all TB patients at the first visit (Figure 3A). Baseline levels of the markers failed to show any significant correlation with each other in individuals with EPTB. Overall, these results suggested that baseline T-Gal9, OPN, and FL-Gal9 levels may be related to the degree of recovery from inflammation in all TB, PTB, and EPTB patients, respectively.

### 3.4. Role of Baseline Inflammatory Markers in Predicting Immunological Recovery in Response to Antituberculosis Treatment

**Figure 5:**
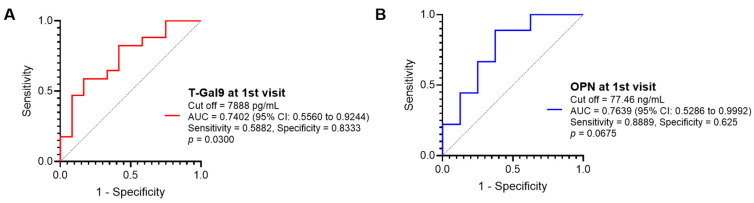
Diagnostic accuracy for discriminating the patients with higher INS from those with lower INS at the first visit. The receiver operating characteristic (ROC) curves are accompanied by cut off values, area under the curve (AUC) with 95% confidence interval (CI), sensitivity, specificity, and *p*-values of (**A**) plasma T-Gal9 levels in all TB patients and (**B**) plasma OPN levels in PTB patients. INS ≥ 6 and <6 are defined as higher and lower scores, respectively, in all TB patients (the median INS is 6 in all TB patients). Similarly, INS ≥ 8 and <8 are defined as higher and lower scores, respectively, in PTB patients (the median INS is 8 in PTB patients).

To confirm whether the measurement of baseline T-Gal9 levels in all TB patients and baseline OPN levels in PTB patients can be used to predict if the patients have higher or lower INS, we performed a receiver operating characteristic (ROC) analysis of these two markers. We determined median INS in each group (median total score of 6 in all TB patients and 8 in only PTB patients), so that we defined the median or higher score as higher INS and the less than the median score as lower INS. As a result, T-Gal9 levels had sufficient accuracy to significantly discriminate higher INS from lower INS (AUC: 0.7402) in all TB patients (Figure 5A). By contrast, OPN levels could not discriminate INS substantially when analyzed in PTB patients (Figure 5B). These results highlighted the potential of T-Gal9 levels as an accurate marker to predict recovery from inflammation in patients with HIV/TB coinfection irrespective of the site of infection.

### 3.5. Role of Early Changes in the Levels of the Inflammatory Markers in Predicting Immunological Recovery in Response to Antituberculosis Treatment

**Figure 6:**
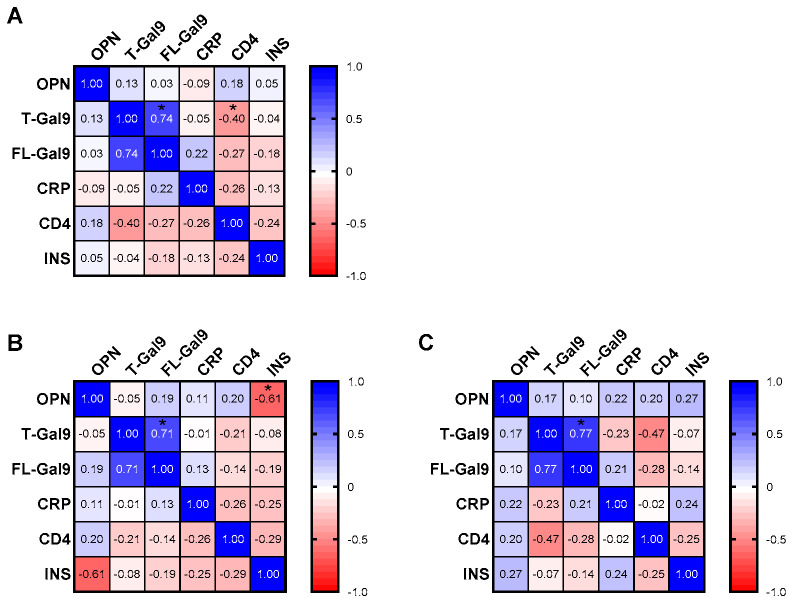
Correlations between the change in each marker and INS in (**A**) all AIDS/TB, (**B**) AIDS/PTB, and (**C**) AIDS/EPTB patients. The first change was calculated by percentage difference in concentration at second visit minus that at first visit. The correlation coefficient is shown in each cell and as colors ranging from blue to red. * *p* < 0.05.

We also examined the correlations of INS with a decrease rate in each marker level at the second visit conducted at 2 months, corresponding to the end of the intensive phase of antituberculosis treatment, to determine if an early decrease in each marker levels can predict immunological recovery at the end of the treatment (Figure 6). A decrease rate in each marker at the second visit was calculated as a percentage of concentration at the first visit minus concentration at the second visit for each marker. It is interesting that the decrease in T-Gal9 levels is associated with an increase of CD4 numbers in all TB patients (Figure 6A). 

**Figure 7:**
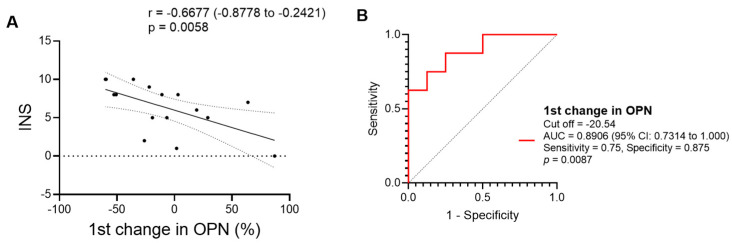
Correlation and discriminating potential of first change in plasma OPN levels with high and low INS in AIDS/PTB. The first change was calculated by percentage difference in concentration at second visit minus that at first visit. (**A**) Scatter plots for negative correlation, accompanied by correlation coefficient (r) with 95% CI and *p*-value. 95% CI is also shown by dotted ranges. (**B**) ROC curves are shown accompanied by cut-off value, AUC value with 95% CI, sensitivity, specificity, and *p*-value. INS ≥ 8 was defined as a higher score and INS < 8 was defined as a lower score (the median INS was 8 in PTB).

INS was positively correlated only with the decrease rate in OPN levels at the second visit in PTB patients (Figure 6B). This correlation was confirmed by the scattered plot (Figure 7A). ROC analysis also showed a sufficient power of the decrease rate in OPN levels at the second visit for discriminating PTB patients with a higher INS from those with a lower INS (Figure 7B).

### 3.6. Chest X-ray (CXR) Severity and Role of Different Markers

**Figure 8:**
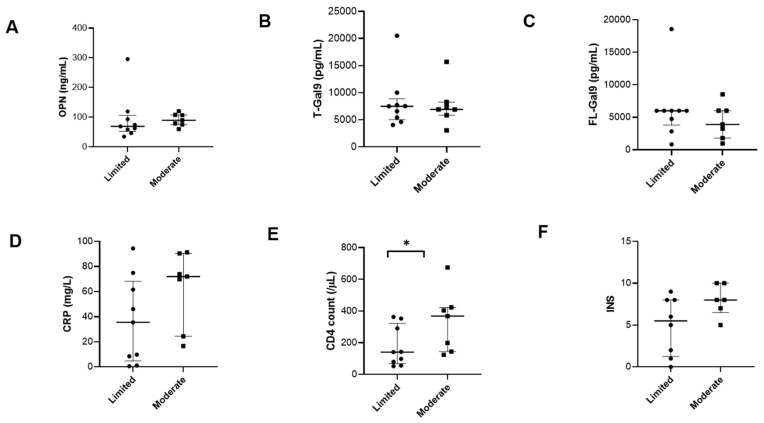
Differences in inflammatory markers with respect to chest X ray severity: Dot plots showing differences in baseline (**A**) plasma OPN levels, (**B**) plasma T-Gal9 levels (**C**) plasma FL-Gal9 levels, and (**D**) plasma CRP levels, as well as (**E**) CD4 count (**F**) and INS, in PTB patients with limited and moderate grade chest X-ray severity. *p* value showing significant change in the CD4 count is indicated as * (*p* < 0.05).

We analyzed CXR grades, baseline levels of inflammatory markers and CD4 data for the CXRs available to us (PTB, n = 16). Limited grade severity was detected in nine patients while moderate grade severity was observed in seven patients. None of the patients had severe grade CXR findings. Although OPN and CRP levels were higher in patients with moderate grade CXRs than those with limited grade, the difference was not significant. Interestingly, a low CD4 count at baseline showed limited lung damage, whereas higher CD4 counts showed moderate lung damage (Figure 8). Gal-9 levels showed no significant difference between the two groups.

### 3.7. Effect of Gender on the Levels of the Different Markers

We evaluated the effects of gender differences on the levels of the markers and other parameters, although female patients were fewer in number than male patients (Table 2). Among men, there seven cases that were ART-naïve and sixteen cases that were on ART were observed. Among women, four cases were ART-naïve and 5 cases were on ART. There is no significant difference between the ratios of males and females according to Fisher’s exact test. Furthermore, there is no significant difference in CD4 counts and viral load between men and women, reflecting the lack of effect of the on–ART ratio. Weight was significantly lower in females as compared to males, as expected. Among the inflammatory markers estimated in the study, only FL-Gal9 levels were observed to be significantly lower in females. There were no significant differences in the levels of the other markers. It is of note that INS was significantly higher in women than men, reflecting better immunologic recovery.

## 4. Discussion

This is the first prospective study monitoring plasma MCP levels in response to ATT in patients with HIV/TB to the best of our knowledge. Previously, we reported increased levels of T-Gal9 and OPN in patients with HIV/TB coinfection using a cross-sectional analytical study [22]. In this study, we first investigated if levels of inflammatory markers such as CRP, T-Gal9, FL-Gal9, and OPN differ in HIV-positive individuals with PTB and EPTB. EPTB represents a milder clinical form of the disease than PTB [25]. PTB and EPTB differ in terms of inflammatory responses, with intense inflammatory changes in PTB, as was also evident from the higher levels of all the inflammatory markers estimated in our study, although significant differences were found only in CRP and FL-Gal9 levels. The Gal9 levels were found to be inversely correlated with CD4 counts, as was also reported previously [15]. Interestingly, our EPTB patients had higher CD4 counts than PTB patients, as a higher proportion of EPTB patients was on ART. This could be an additional reason for the significantly lower FL-Gal9 levels in our EPTB patients. CRP levels have mainly been evaluated as a screening tool for diagnosing PTB, and data comparing the levels in HIV-positive individuals with PTB and EPTB are sparse. One of the studies reported a cut-off of 8.25 mg/L for diagnosing PTB as well as EPTB in HIV-positive individuals [26]. However, more than half of the individuals with EPTB in our study had CRP levels lower than this cut-off, making the utility of CRP levels in diagnosing EPTB in HIV-positive individuals questionable. We also investigated the effect of antituberculosis treatment on the levels of these four markers in this time course analytical study. We showed that CRP, T-Gal9, FL-Gal9, and OPN decreased significantly in the PTB type, but only FL-Gal9 showed a significant decrease in the EPTB type. Thus, significantly decreased levels of FL-Gal9 in successfully ATT-treated individuals with EPTB and PTB were observed, suggesting the role of FL-Gal9 as a marker for monitoring response to the treatment of patients with HIV/TB coinfection, irrespective of the site of the infection. 

Apart from monitoring the study participants clinically and microbiologically to detect treatment success, we also followed them up to determine immunological improvement. The TB patients showed excessive immune activation which subsided upon treatment. Since we enrolled patients with HIV, it is important to determine the persistence of immune activation, which might point to the persistence of the infectious etiology, further leading to AIDS- or non-AIDS-related events. Immunological monitoring was carried out by calculating INS, reflecting the subsidence of the inflammatory response by monitoring changes in the levels of OPN, Gal-9, and CRP. It is of note that T-Gal9, but not FL-Gal9, positively correlated with immunologic recovery in all TB patients at the first visit. We previously demonstrated that the FL-Gal9 is cleaved by proteases and the blood levels of a mixture of full-length and cleaved products reflect the severity of HIV/OI, and pointed that FL-Gal9 and Tr-Gal9 levels have different responses to pathological conditions [13]. In fact, Gal-9 proteolysis is regulated by HIV infection in MTB patients in relation to MMP-9 activity [27]. Our results indicated that higher levels of T-Gal9 at the first visit predicted improvements in inflammatory responses. These findings may be related to the fact that Gal-9(+) Th cells as well as exogenous Gal-9 regulate Th17/Treg development [28]. In contrast to T-Gal-9, an inverse correlation of FL-Gal9 levels with INS was observed in EPTB, indicating that FL-Gal9 could serve as a marker of therapeutic efficacy in EPTB types. We reported that the NCRD and CCRD of Gal-9 show more potent and different immunomodulating activities than FL-Gal9 [29]. This difference in the functionalities could have led to the differing correlations observed in our study. 

The administration of recombinant Gal-9 was shown to modulate immunity bidirectionally, not only suppressing excessive immunity and inflammation but also enhancing these functions in the context of compromised immunity [30]. Gal-9 was reported to bind to Tim-3 and induce a signaling pathway that is known to suppress the generation of Th17 cells in acute renal injury in mice [31]. On the other hand, it was also found that Gal-9 activates human natural killer cells and induces interferon-gamma release via CD44 molecules [32]. Low plasma Gal-9 levels were associated with a better response in rheumatoid arthritis [33], while in cervical cancer, the presence of Gal-9 was associated with a better prognosis regarding overall survival [34]. Our findings suggest that T-Gal9 levels at the first visit could reflect the immunomodulating activity of Gal-9, leading to the use of T-Gal9 levels to predict the recovery of inflammation (high INS) in HIV/TB. FL-Gal9 levels, which represent intact Gal9, could be deleterious for immunological recovery, especially in the context of EPTB. Further immuno-biochemical analysis would be necessary to investigate which pathological conditions favor the production of Gal-9 in EPTB, because Gal-9 could be produced by a variety of cells [35]. We have shown the diffuse expression of Gal-9 in TB granuloma in peritoneal TB patients and huge amounts of Gal-9 in pleural fluids [36]. Neutrophils are also important mediators of innate immune response in TB infection [37] and are good reservoirs of Gal-9 [38]. In EPTB, lymphatic systems are the most frequently involved, but disseminated infection and central nervous system involvement is also often seen in immunocompromised hosts [39]. The production of Gal-9 in endothelium cells is reported to promote leukocyte recruitment in mice, indicating that Gal9 could reflect systemic inflammation [40]. Diagnostic methods for various types of EPTB using tissues and body fluids have been developed [41], and searching for the Gal-9 metabolism in EPTB would be useful for diagnosis.

OPN levels were significantly decreased in PTB, but not in EPTB. At the first visit, OPN levels predicted high INS, implying immunologic recovery only in PTB. OPN was proposed to play a role in granuloma formation [42]. The level of OPN protein expression was inversely correlated with disseminated infection, ill-defined granulomas, and the patient’s death [19]. Significantly higher levels of OPN in PTB than EPTB in HIV-negative individuals were reported by us previously, indicating the role of OPN in the containment of TB via mediating its protective as well as immunopathogenic effects, predominantly in PTB, through granuloma formation. Recently, gene therapy using adenovirus vector-encoding OPN led to high immune responses against TB infection in mice, suggesting its potential as a co-adjuvant in treating PTB [20]. In PTB, the decrease rate in OPN levels after two months of therapy also reflected a higher INS value, indicating an OPN decrease, after two months, which reflects the final therapeutic effect. In other words, a contradictory phenomenon can be seen, in which a high OPN predicts a high value of INS at the first visit, and a further decrease in OPN predicts a high value of INS at the second visit. The decrease rate at the second visit may reflect the therapeutic efficacy. These bidirectional activities of OPN may also be induced by either FL-OPN or cleaved OPN, because both were detected as an OPN in this study using R&D Elisa [24]. Thrombin cleaved OPN is known to bind to an integrin different from FL-OPN and to cause anti-type II collagen antibody-induced arthritis [43]. The C-terminal domain of OPN binds to CD44 and induces macrophage chemotaxis, and a non-overlapping N-terminal OPN domain binds to beta(3)-integrin receptors and induces cell spreading and subsequent activation [44]. Very recently, it was proposed that the thrombin cleavage of OPN facilitates tumor progression and suppresses host anti-tumor immune responses [45]. Both cleaved and FL-OPN levels are elevated in the blood of various infectious diseases [14]. We need to clarify the pathological roles of both FL and cleaved OPN by measuring each of the two different types of OPN in PTB patients.

CD4T cells protect against MTB infection because the presence of CD4 molecules on CD1 b-restricted T cells leads to higher tetramer binding than negative cells [46]. In HIV/TB coinfection, however, the classic picture of PTB is seen in patients with relatively higher CD4 counts rather than in those with severe immunocompromised conditions [7]. We observed that the patients with moderate CXR findings showed significantly higher CD4 cell numbers than mild cases. A higher CD4 count may imply better immune reconstitution, facilitating enhanced inflammatory response and leading to increased lung damage in PTB patients. HIV/TB patients with CD4 counts <150 cells·μL^−1^ were shown to be five times more likely to have a normal chest radiograph than HIV-negative TB patients [47]. Moreover, the ART-mediated reversal of CD4 lymphopenia in advanced HIV/TB patients was associated with incident lung involvement in one of the studies [48]. CD4 T-cells are thought to mediate excessive inflammation and subsequent lung damage by secreting cytokines like TNF-α and IFN-γ and activating effectors like MMPs [49]. This is also a likely reason for the association of cavitation, which represents extensive lung damage with higher CD4 cell counts, as reported in various studies [8,50]. Further, CD4T cells also play an important role in mediating the immune reconstitution inflammatory syndrome in HIV/TB coinfection through the exacerbated immune responses mounted by them against mycobacterial antigens, stressing their roles in mediating lung damage [51]. 

We searched for the role of gender differences in the levels of inflammatory markers and immunologic recovery. In India, the risk of death in male HIV-positive patients was 1.24 times higher than that of female patients. Furthermore, the survival probabilities were higher in female patients compared with male patients when followed-up for a period over 7 years [52]. The survival benefit of female gender has also been demonstrated in patients with HIV/TB coinfection [53]. The reasons for the favorable prognosis of female patients are not clear, but our analysis provides a novel aspect to consider regarding female patients, because the INS is higher in female patients. These differences are not due to on-ART rates, as these are no different between male and female patients. In addition, the higher FL-Gal9 levels in male patients may explain the poor outcome in males because the FL-Gal9 levels are inversely correlated with INS in EPTB. The reasons for the lack of difference between males and females in terms of T-Gal9 levels are unclear. As far as we know, there is no paper that reported gender differences in FL-Gal9 levels, probably because the studies measured T-Gal9 in most cases. In relation to these findings, estradiol + medroxyprogesterone acetate treatment enhances LGALS9 mRNA expression in endometrial stroma cells, indicating that sex hormones affect the metabolism of Gal-9 [54]. We believe that gender differences in FL-Gal9 should be further investigated to understand the pathological roles of Gal-9, as well as to devise personalized management strategies focusing on gender differences.

There are some limitations to this study. The number of patients was not enough. Although OPN and Gal-9 are elevated in various infectious diseases [13,14], we could not rule out other coexisting infections, like cryptococcosis, cytomegalovirus, hepatitis B, and hepatitis C. Also, it was not possible to confirm all cases of EPTB microbiologically, as the collection of samples was not possible for all the cases.

## 5. Conclusions

Our study evaluated the pathological roles of MCPs in patients with HIV/TB coinfection undergoing ATT. Four markers, OPN, T-Gal9, FL-Gal9, and CRP, decreased significantly in PTB patients, with FL-Gal9 showing the biggest decrease. In EPTB patients, only FL-Gal9 was significantly decreased at the end of therapy, and FL-Gal9 levels at the second visit serve as a marker of therapeutic efficacy (high INS), indicating its role in monitoring treatment success in EPTB patients irrespective of the site of infection. The positive association of the baseline value of T-Gal9 with the INS in all TB patients suggested its protective role in mediating recovery from inflammation in TB patients. In PTB, the baseline value of OPN was positively associated with INS, while the decrease rate in OPN levels at the second visit positively correlated with INS, suggesting a bidirectional role of OPN, mainly in PTB patients. The improvement in CRP levels did not show any such predictive power. It would be interesting to evaluate the associations between INS and the long-term effects of antituberculosis treatment in HIV/TB coinfection in terms of TB recurrence and the occurrence of other AIDS/non-AIDS events. The study also indicated a need to further evaluate the role of gender differences in FL-Gal9 levels in mediating the immunopathology of TB infection. In the future, we need to consider if monitoring immunologic recovery will contribute to better patient management.

## Figures and Tables

**Table 1:** Profiles of the patients.

Parameters, Median (IQR)	PTB	EPTB	All TB	*p* Value
	n = 20	n = 12	n = 32	
Age (Years)	46 (40–52)	40.5 (33–48)	45.5 (38–50)	0.1431
Body weight (kg)	46.7 (41–56)	49.5 (38–64)	48.5 (40–56)	0.7228
Duration of therapy (months)	6 (6–12)	6 (6–12)	6 (6–12)	0.8956
Gender:Male:Female	14:06	09:03	23:09	>0.9999
CD4 count (cells/μL)	181.5 (128–360.5)	383 (325–528.8)	143 (304–399.8)	**0.0055**
≤200 no. (%)	11 (55)	1 (8.3)	12 (37.5)	-
201–500 no. (%)	8 (40)	7 (58.3)	15 (46.9)	-
>500 no. (%)	1 (5)	4 (33.3)	5 (15.6)	-
Viral Load (copies/mL),	155.5 (0–65,547)	0 (0–31,204)	0(0–54,508)	0.2014
INS	8 (5–9.75)	5 (3–7)	6 (5–8)	0.1024
OPN (ng/mL)	91.7 (64.1–114.4)	87.5 (71.8–100.5)	89.6 (69.4–107.6)	0.833
T-Gal9 (pg/mL)	7473 (6056–9095)	6593 (4099–10,952)	7473 (4689–9862)	0.7445
FL-Gal9 (pg/mL)	6000 (3409–6000)	3502 (1274–5604)	5371 (2895–6000)	**0.0305 (one tailed)**
CRP (mg/L)	53.9 (9.6–74.7)	8.0(2.9–20.0)	19.9 (6.8–71.5)	**0.0081**

Bold *p* values indicate statistical significance. IQR: interquartile range; TB: tuberculosis; PTB: pulmonary TB; EPTB: extrapulmonary TB; INS: inflammatory score; OPN: osteopontin; T-Gal9: total galectin-9; FL-Gal9: full-length galectin-9; CRP: C-reactive protein.

**Table 2:** Genders and markers.

Parameters Median (IQR)	Male (n = 23)	Female (n = 9)	*p* Value
PTB:EPTB	14:09	06:03	NS
ART naïve (%)	30.4	44.4	NS
Weight (Kg)	52.4 (43.9–80)	38.25 (33–70.9)	**0.0042**
CD4 (cells/μL)	291 (141–569)	317 (180.3–794)	NS
Viral load (copies/mL)	0 (0–376,409)	150 (0–331,792)	NS
OPN (ng/mL)	90.3 (69.0–307.7)	86.3 (73.4–224.5)	NS
T-Gal9 (pg/mL)	7439 (4657–20,539)	9512 (6281–15,706)	NS
CRP (mg/L)	20.18 (6.664–94.44)	16.72 (4.936–90.48)	NS
FL-Gal9 (pg/mL)	6000 (3855–18,573)	3197 (906–6000)	**0.0353**
INS	5 (3–10)	9 (8–10)	**0.0009**

Bold *p* values indicate statistical significance. IQR: interquartile range; TB: tuberculosis; PTB: pulmonary TB; EPTB: extrapulmonary TB; INS: inflammatory score; OPN: osteopontin; T-Gal9: total galectin-9; FL-Gal9: full-length galectin-9; CRP: C-reactive protein; NS: not significant.

## Data Availability

All data are available upon request.
